# Causes and Consequences of Interindividual Response Variability: A Call to Apply a More Rigorous Research Design in Acute Exercise-Cognition Studies

**DOI:** 10.3389/fphys.2021.682891

**Published:** 2021-07-22

**Authors:** Fabian Herold, Alexander Törpel, Dennis Hamacher, Henning Budde, Liye Zou, Tilo Strobach, Notger G. Müller, Thomas Gronwald

**Affiliations:** ^1^Department of Neurology, Medical Faculty, Otto von Guericke University, Magdeburg, Germany; ^2^Research Group Neuroprotection, German Center for Neurodegenerative Diseases (DZNE), Magdeburg, Germany; ^3^German Swimming Federation, Kassel, Germany; ^4^Department of Sport Science, German University for Health and Sports (DHGS), Berlin, Germany; ^5^Faculty of Human Sciences, MSH Medical School Hamburg, Hamburg, Germany; ^6^Exercise and Mental Health Laboratory, Institute of KEEP Collaborative Innovation, School of Psychology, Shenzhen University, Shenzhen, China; ^7^Department of Psychology, MSH Medical School Hamburg, Hamburg, Germany; ^8^Center for Behavioral Brain Sciences (CBBS), Magdeburg, Germany; ^9^Department of Performance, Neuroscience, Therapy and Health, Faculty of Health Sciences, MSH Medical School Hamburg, Hamburg, Germany

**Keywords:** exercise prescription, interindividual variability, intraindividual variability, cognition, neuroscience, acute physical exercise, physical activity

## Abstract

The different responses of humans to an apparently equivalent stimulus are called interindividual response variability. This phenomenon has gained more and more attention in research in recent years. The research field of exercise-cognition has also taken up this topic, as shown by a growing number of studies published in the past decade. In this perspective article, we aim to prompt the progress of this research field by (i) discussing the causes and consequences of interindividual variability, (ii) critically examining published studies that have investigated interindividual variability of neurocognitive outcome parameters in response to acute physical exercises, and (iii) providing recommendations for future studies, based on our critical examination. The provided recommendations, which advocate for a more rigorous study design, are intended to help researchers in the field to design studies allowing them to draw robust conclusions. This, in turn, is very likely to foster the development of this research field and the practical application of the findings.

## Introduction

Every human is unique, and every day is different. Hence, it is not surprising that a certain degree of variability is present in measures of human capacity and performance even though the assessment is conducted during apparently similar conditions. From a scientific point of view, different origins of variability can be distinguished, namely (i) technical variability (also known as measurement error; e.g., due to difference in machine calibration), (ii) intraindividual variability (also known as within-subject variability), and (iii) interindividual variability (also known as between-subject variability) (Hecksteden et al., 2015; Voisin et al., 2019). Especially, the phenomenon of interindividual variability has gained more and more attention in recent years. Among others, this increased interest was driven by the intervention literature because the intervention-related individual differences in outcome measures (e.g., neurocognitive parameters) have great practical relevance (e.g., in therapy, rehabilitation, health care, prevention, and sports medicine) (Greenham et al., 2018; Ross et al., 2019).

The research on interindividual differences has currently reached the field of exercise-cognition research as illustrated by recently published studies investigating interindividual variability in cognitive measures in response to acute bouts of physical exercises (Yamazaki et al., 2017, 2018; Schwarck et al., 2019). In this context, physical exercise is defined as a specific form of physical activity that is planned, structured, repetitive, and purposive to maintain or improve a certain outcome, for instance, physical or cognitive parameters. In contrast, physical activity itself comprises all (unspecific) muscle-induced bodily movements leading to an increase in energy expenditure above ∼1.0/1.5 metabolic equivalent of task [MET; 1 MET = 1 kcal (4,184 kJ) • kg^−1^ • h^−1^] (Caspersen et al., 1985). Thus, physical exercise is always physical activity but physical activity is not necessarily physical exercise (Wegner et al., 2020). In this perspective article, we focus on physical exercise, particularly on an acute (single) bout of physical exercises rather than on the effects of chronic exercises (e.g., repeated bouts of physical exercises over a longer period of time) or physical training [e.g., chronic physical exercises being conducted regularly in a planned, structured, and purposive manner with the objective of increasing or, at least, maintaining one or more fitness components (Scheuer and Tipton, 1977; Caspersen et al., 1985; Howley, 2001)]. Acute physical exercises are a good starting point to investigate interindividual variability in neurocognitive outcomes since a lower amount of resources is required (e.g., financing, equipment, and personnel) as compared to long-term studies. Moreover, the knowledge and experience gained by studies investigating interindividual variability in response to acute physical exercises can in a later step be helpful to conceptualize physical training studies that are more challenging to implement. However, it should be noted that our recommendations can, at least partly, also be transferred to chronic physical exercises or physical training.

In our opinion, future research in the direction of interindividual variability in response to an acute bout of physical exercise can greatly benefit from the application of more rigorous study designs. Based on a brief discussion of the origin of interindividual variability and the analysis of available studies, we will deduce practical recommendations for future studies aiming to investigate interindividual differences in specific outcome parameters in response to acute physical exercises.

## The Complex Story of Influencing Factors and Their Consequences

**Figure 1:**
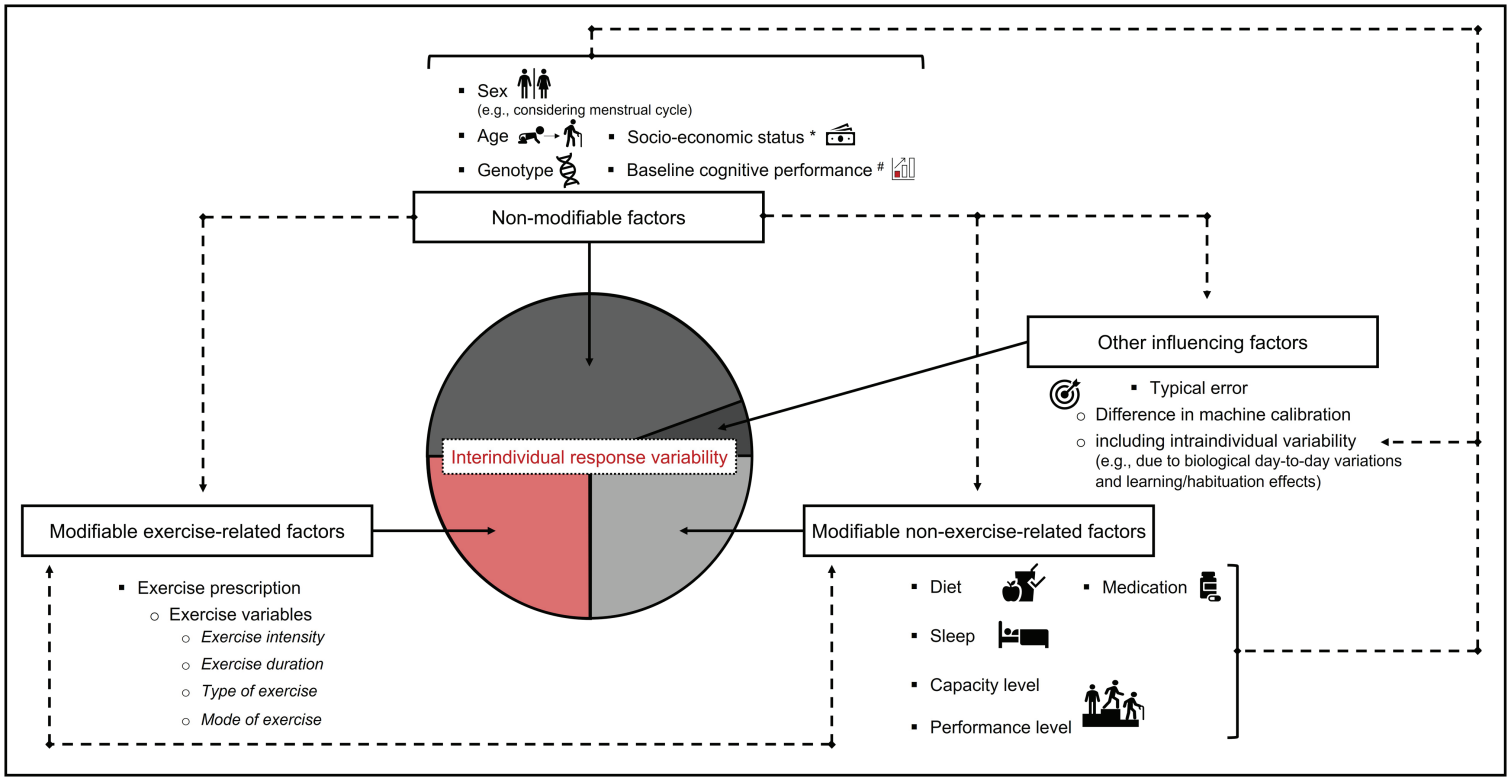
Schematic illustration of the influence of non-modifiable factors, modifiable factors, and other influencing factors that causes interindividual response variability in neurocognitive outcomes after an acute bout of physical exercises. Please note that some factors contributing to interindividual response variability mutually influence each other and that the used ratio of the segments presented in the pie chart does not necessarily reflect the actual proportion of their influence. Dashed lines indicate that these factors have an influence on other factors. Capacity level is defined as the actual or potential ability of an individual (e.g., athlete) to accept load. The capacity level is influenced by various factors, including fitness status before load, loading rate, psychological factors, and various other internal and external factors (Soligard et al., 2016). Here, we included performance level as fitness status, as a specific domain of capacity in an organismic subsystem or an external load outcome parameter. ^*^Socio-economic status including factors such as (parental) income, (parental) education, and (parental) occupation. ^#^Baseline cognitive performance refers to the baseline performance capabilities of individuals with respect to a specific cognitive task that can be, among other factors, influenced by perceptual skill level, socioeconomic status (SES), and the global cognitive performance. Please note that socio-economic status and baseline cognitive performance are not completely non-modifiable but certain aspects cannot be changed in later life periods (e.g., developmental trajectories influenced by parental income and parental education that shaped cognitive performance in adulthood).

There are ongoing efforts to understand the factors that cause exercise-related interindividual variability in specific outcome parameters (Ross et al., 2019). In recent years, a growing amount of research has emerged showing that there is considerable interindividual variability in changes of cardiorespiratory fitness [operationalized *via* maximal or highest “system-limited” oxygen uptake (VO_2MAX_; VO_2PEAK_)] in response to physical training (Karavirta et al., 2011; Bonafiglia et al., 2016; Gurd et al., 2016; Williamson et al., 2017; Bratland-Sanda et al., 2020; Metcalfe and Vollaard, 2021). Here, for example, Karavirta et al. (2011) reported that changes in VO_2MAX_ can range between −8 and 42% in older adults who had participated in a combined strength and endurance training program over 21 weeks. In other words, this research has demonstrated that there is a wide range of response levels to an apparently identical exercise stimulus with some individuals benefitting more than others from physical training regimes. As shown in Figure 1, several factors constitute a potential source for interindividual response variability (e.g., neurocognitive changes in response an acute bout of physical exercises). According to the literature, these factors can be categorized as follows: non-modifiable, modifiable, and other influencing factors.

### Non-modifiable Factors

Non-modifiable factors comprise factors that are predetermined, such as genetics, sex, and age. Consequently, these factors cannot be manipulated experimentally, but acknowledging their influence on exercise-related responses, researchers can account for them when designing studies. Based on this, it can be determined to which extent the observed interindividual variability in a specific (neurocognitive) outcome parameter is caused by the difference in the individual response to the physical exercise itself or is attributable to other factors, such as non-modifiable factors.

There is considerable evidence highlighting the prominent influence of the *genotype* on the responsiveness of a single individual in (i) physical performance parameters (Bouchard et al., 1999, 2011, 2012, 2015; Timmons et al., 2010; Puthucheary et al., 2011; Timmons, 2011; Costa et al., 2012; Pickering and Kiely, 2017; Sarzynski et al., 2017; Williams et al., 2017; Alvarez-Romero et al., 2020), (ii) brain structure (Thompson et al., 2001; Toga and Thompson, 2005; Bueller et al., 2006; Peper et al., 2007; Yoon et al., 2010), (iii) brain function (Anokhin et al., 2006; Blokland et al., 2008; Makris et al., 2009; Glahn et al., 2010), and (iv) cognition (McClearn, 1997; Anokhin et al., 2003; Goldberg and Weinberger, 2004; Blokland et al., 2008; Friedman et al., 2008). Undoubtedly, the gain of knowledge in this direction will help us to better tailor the exercise prescriptions according to an individual’s characteristics (Herold et al., 2019, 2020c; Barha et al., 2021a). However, the exact influence of genetic factors on interindividual response variability, at least for physical performance, is not yet exactly known and is currently under debate (Joyner and Lundby, 2018; Bouchard, 2019; Joyner, 2019a,b). In this context, there is some evidence showing that genetic factors (e.g., specific gene variants) predict a certain degree of variance in the training adaptations with regard to a specific outcome parameter (e.g., VO_2MAX_; Bouchard et al., 1999, 2011; Timmons et al., 2010; Bouchard, 2012, 2019). While the variance explained by genetic variants has a relatively wide range (e.g., from 22 to 57% for VO_2MAX_; Zadro et al., 2017), some of these genetic variants have been frequently associated with variance of an outcome parameter (e.g., VO_2MAX_) across different studies (for review, see Alvarez-Romero et al., 2020). However, there is limited evidence linking those genetic factors to key physiological pathways being causal to drive adaptations (e.g., changes in stroke volume; Joyner and Lundby, 2018; Joyner, 2019a,b). This challenging the overemphasis and reductionism of interindividual variability to genetic factors. Thus, further studies in this direction should consider multiple levels of analysis, including a multiscale physiological responses approach, to better understand physical exercise-related responses in general and the contribution of genetic factors to them in particular (Herold et al., 2019; Joyner, 2019a,b). In this regard, twin studies in mono- and dizygotic twins have been proposed as a valuable option to investigate the influence of genetic factors and/or exercise prescription on specific outcome parameters (Joyner and Lundby, 2018).

Concerning acute exercise-cognition studies, there is, to the best of our knowledge, no twin study available, and the impact of genetic factors on interindividual variability in neurocognitive outcome parameters in response to acute physical exercises has been, so far, not extensively investigated. Notably, one study suggested that genetic factors (e.g., BDNF genotype) might influence memory performance (Piepmeier et al., 2019). However, given the small sample size of the mentioned study, no detailed analysis of interindividual response variability could be performed. Thus, additional studies with larger sample sizes are needed to extend the knowledge in this direction.

Another non-modifiable factor is *sex*. There are not many studies that investigated its role in the acute exercise-cognition field (Loprinzi and Frith, 2018), but it is assumed that sex can be an important moderator especially in long-term physical exercise interventions (Barha et al., 2017a,b, 2019, 2021b; Barha and Liu-Ambrose, 2018, 2020). The available studies that investigated the influence of sex on cognitive performance after an acute bout of physical exercises did not find compelling evidence that sex moderates the effects on memory performance at the group level (Johnson et al., 2019; Johnson and Loprinzi, 2019). However, the answer to the question whether these findings can be generalized to the individual level also requires further investigations.

There is some evidence from meta-analyses that *age* is also an important factor influencing the magnitude of the effect of acute exercise on cognitive performance at the group level and should not be disregarded (Chang et al., 2012; Ludyga et al., 2016; Oberste et al., 2019). In this respect, it was observed that preadolescent children and older adults benefit from acute physical exercises more than other age groups (Ludyga et al., 2016). In addition, there is also evidence, albeit limited, that exercise-induced changes in functional brain activation patterns are influenced by age (Yu et al., 2021). However, the effect of age on interindividual differences in response to acute physical exercises in terms of cognitive performance has not yet been sufficiently studied in order to find a physiologically based explanation for the mentioned phenomenon.

In addition, there is some evidence that non-modifiable factors such *socioeconomic status* (SES) can influence the brain integrity and cognitive performance (Hackman and Farah, 2009; Hackman et al., 2010; Staff et al., 2012; Zhang et al., 2015; Farah, 2017; Cermakova et al., 2018; Chan et al., 2018; Korous et al., 2020; Steptoe and Zaninotto, 2020; Yaple and Yu, 2020). Although the direct influence of SES on interindividual variability of cognitive outcomes has, to the best of our knowledge, not extensively examined in acute-exercise cognition studies, it is a crucial factor that probably contribute, among other factors, to interindividual variability in a specific neurocognitive outcome parameter and thus should be assessed in future studies investigating interindividual variability.

As shown in Figure 1, *baseline cognitive performance* is an important non-modifiable factor based on the observations that it influences the effects of acute physical exercises on cognitive performance (Drollette et al., 2014; Yamazaki et al., 2018; Ishihara et al., 2020). In the above-mentioned studies, it was noticed that individuals with a low baseline performance benefit the most from the acute physical exercise interventions in terms of cognitive performance enhancements (Drollette et al., 2014; Yamazaki et al., 2018; Ishihara et al., 2020). However, concerning interindividual variability in baseline cognitive performance, the statistical phenomenon of *regression to the mean (RTM)* should be considered (Shephard, 2003; Chiolero et al., 2013; Atkinson and Batterham, 2015). RTM describes the phenomenon that in studies with several measurement time points (i.e., repeated-measures design), extreme values being measured at the baseline assessment tend to be closer to the mean (i.e., less extreme value) in a follow-up assessment (for a more detailed discussion, see Shephard, 2003; Barnett et al., 2005; Chiolero et al., 2013; Atkinson and Batterham, 2015). Theoretically and in line with the phenomenon of the RTM, individuals with a low level of cognitive performance at baseline tend to show larger improvements at the second assessment after an intervention (e.g., after an acute bout of physical exercises), whereas individuals with a high level of cognitive performance at baseline tend to show smaller improvements at the second assessment after an intervention (e.g., an acute bout of physical exercises). To account for the phenomenon of RTM, a proper study design and sophisticated analysis methods are necessary (see recommendations in section *Considerations regarding the statistical analysis*; Shephard, 2003; Barnett et al., 2005).

#### Recommendations Regarding Non-modifiable Factors

The currently available evidence suggests that some non-modifiable factors can mediate the effects of acute physical exercises on cognitive performance (at least at the group level) but whether these factors contribute to the interindividual response variability has not yet been extensively investigated. Hence, future research in this direction is urgently needed. Taking non-modifiable factors into account (e.g., age and sex) is relatively easy, as these factors can be considered during the recruitment of participants or can be used as covariates in the statistical analysis. In contrast, the factor genetics is more complex and needs interdisciplinary efforts to be appropriately addressed (e.g., collaboration between exercise scientists and geneticists). However, one should always keep in mind that non-modifiable factors are factors that we have to deal with, for instance, by adjusting the modifiable factor exercise prescription (Lightfoot, 2008; Herold et al., 2019, 2020c; Gronwald et al., 2020b).

### Modifiable Factors

Modifiable factors can be classified into exercise-related and non-exercise-related factors. Exercise-related factors include parameters of exercise prescription (e.g., exercise intensity), whereas non-exercise-related factors comprise lifestyle and personal factors (e.g., sleep and diet) which can affect (in a positive or negative way) the acute and/or chronic response to physical exercise. In the following part, we want to address both factors more in detail.

#### Modifiable Exercise-Related Factors

In the literature, there is empirical evidence (Ross et al., 2015; Bonafiglia et al., 2016; Montero and Lundby, 2017) and a strong theoretical rationale (Pickering and Kiely, 2018; Herold et al., 2019; Gronwald et al., 2020b; Herold et al., 2020c; Meyler et al., 2021) that the individual responsiveness to exercise can be influenced by modifying the dose of exercise by adjusting the exercise prescription. However, a recent article emphasizes that larger mean changes rather than the reduction in interindividual variability, are responsible for the better responsiveness after adjusting the exercise prescription (e.g., higher training frequency, longer training duration) (Bonafiglia et al., 2021b). The latter finding might indicate that a more rigorous individualization is needed to decrease interindividual variability (as suggested by Herold et al., 2019, 2020c). However, our knowledge regarding the influence of an adjusted exercise prescription on neurocognitive outcome parameters is meager. In this context, there are findings showing that exercise prescription of acute physical exercise [e.g., exercise intensity, mode of exercise protocol (continuous vs. interval training), or type of exercise (physical exercise with or without motor/cognitive activities)] influences neurocognitive parameters such as cognitive performance at the group level (Budde et al., 2008, 2010; Lambourne and Tomporowski, 2010; Chang et al., 2012; Netz, 2019; Oberste et al., 2019, 2021; Pontifex et al., 2019) or functional brain activity patterns (Mehren et al., 2019). Moreover, there is a good theoretical rationale that the exercise prescription influences the interindividual response variability of neurocognitive outcomes (Herold et al., 2019). However, to the best of our knowledge, no empirical study has adequately addressed this point.

Recently published studies, which focused on the interindividual variability of cognitive outcomes to acute physical exercises, have not comprehensively addressed these issues. Hence, proper results to derive new insights for this field of science are missing. For instance, the study of Schwarck et al. (2019) applied two types of running exercise protocols including moderate-intensity continuous exercise (MICE) at 40–59% VO_2MAX_ and high-intensity interval exercise (HIIE; 5 × 2 min intervals at 90% VO_2MAX_ interspersed with 3 min active recovery at 40% VO_2MAX_). By using both different exercise intensities and different modes of exercise protocols (MICE and HIIE), not only the exercise intensity (as only discussed by the authors) but also the mode of exercise is different (Jiménez-Pavón and Lavie, 2017a,b; Jiménez-Pavón et al., 2019). Thus, a physical exercise intervention including more than one modification on exercise prescription does not allow to infer which independent variable (i.e., intensity vs. mode) has influenced the dependent variable (e.g., acute changes in cognitive performance) and thus no comprehensive insights can be obtained with regard to a dose-response relationship.

#### Recommendations Regarding Modifiable Exercise-Related Factors

In summary, unarguably more well-designed studies are needed to appropriately address the question of how the adjustment of exercise prescription influences the interindividual variability in neurocognitive outcome parameters in response to acute physical exercises (Herold et al., 2019, 2020b,c).

A crucial point to rule out the influence of exercise-related factors on interindividual variability of neurocognitive outcomes is to appropriately define and report the exact exercise prescription. In this context, we strongly recommend to report parameters of external and internal load to characterize the exercise regime (Gronwald et al., 2019; Herold et al., 2019, 2020a,c). Adhering to this recommendation will allow for a better comparison across different studies (Gronwald et al., 2019; Herold et al., 2020a,c). In this context, *external load* is characterized as the work that an individual performs regardless of internal characteristics, whereas the *internal load* is defined as the individual and acute psychophysiological response (e.g., physiological, psychological, motor, and biomechanical responses) to the external load. This response is influenced by lifestyle (e.g., diet and sleep) and environmental factors (e.g., climate and equipment) (Wallace et al., 2009; Halson, 2014; Soligard et al., 2016; Bourdon et al., 2017; Burgess, 2017; Vanrenterghem et al., 2017; McLaren et al., 2018; Impellizzeri et al., 2019). In the context of exercise prescription, the external load should be carefully adjusted to obtain an interindividually comparable internal load which is important to achieve a certain “dose” (Gronwald and Budde, 2019; Herold et al., 2019, 2020b,c; Gronwald et al., 2020b).

All available studies which investigated interindividual differences in cognitive performance in response to an acute bout of physical exercises based their exercise prescription on specific percentages of VO_2MAX_ (Schwarck et al., 2019), respectively VO_2PEAK_ (Yamazaki et al., 2017, 2018). However, it is still a matter of an ongoing debate which is the most suitable parameter to prescribe exercise intensity in endurance exercises (Hofmann and Tschakert, 2010; Tschakert and Hofmann, 2013; Jamnick et al., 2020), and this debate has recently reached the field of exercise-cognition research, too (Gronwald et al., 2018, 2019; Suwabe et al., 2018; Herold et al., 2019, 2020b). In course of this debate, a strong theoretical rationale has been developed, suggesting that alternative approaches of exercise prescription should be tested and applied in the field of exercise-cognition research (Herold et al., 2019, 2020b). In particular, it was hypothesized that an exercise intensity prescription (considering influencing factors such as exercise-specific adaptations and performance level), which uses the individual lactate-level (Herold et al., 2019) or the individual brain oxygenation pattern (Herold et al., 2020b) to gauge exercise intensity, can lead to valuable insights regarding interindividual differences in cognitive performance in response to acute physical exercises.

In brief, although this perspective article cannot discuss all possible approaches of exercise (intensity) prescription in full detail, we want to stress that the current gold-standard methods of exercise intensity prescription which are based on fixed-percentages of maximal values [e.g., VO_2MAX_ or maximal heart rate (HR_MAX_)] cannot be recommended unreservedly because they can lead to high interindividual variability in other internal load parameters (e.g., lactate) (Hofmann and Tschakert, 2010; Tschakert and Hofmann, 2013; Herold et al., 2019; Iannetta et al., 2020; Jamnick et al., 2020). Thus, we strongly advocate for the investigation of alternative approaches of exercise intensity prescriptions in the field of exercise-cognition science (Herold et al., 2019, 2020b). Based on recent overview articles on this topic (Mann et al., 2013; Jamnick et al., 2020; Meyler et al., 2021), we recommend to use submaximal anchors of respiratory or/and metabolic parameters (e.g., ventilatory or lactate thresholds) as a proxy of aerobic and anaerobic threshold marker for exercise intensity prescription. A limitation of threshold concepts, which needs to be acknowledged, is that depending on the intensity domain, some of the threshold concepts are more valid than others (Jamnick et al., 2020). In addition, there is a strong theoretical basis for the application of these submaximal threshold concepts to prescribe exercise intensity in endurance-type exercises. However, the challenges and pitfalls in determining these individual thresholds (e.g., the need of verification of threshold markers in two to three additional visits) may explain why many researchers continue to favor exercise intensity prescriptions based on fixed percentages of maximum values (Hofmann and Tschakert, 2010; Mann et al., 2013; Herold et al., 2019). Hence, the effort of including threshold-based concepts may generate the same individual variation in internal load parameters as the prescription with fixed-percentage of maximum values when no additional verification is performed (Mann et al., 2013).

In conclusion, none of the methods for exercise intensity prescription is without limitations. The most appropriate concept for acute exercise-cognition in our view is to include multiple factors (e.g., exercise intensity domain, number and characteristics of participants, and study resources) and to find a compromise between scientific best practice recommendations and practical constraints (e.g., study resources and/or technology; Mann et al., 2013). In this context, recently introduced approaches which are based on system dynamic interactions and the analysis of non-linear features of heart rate variability appear to be a promising alternative to traditional approaches as they enable real-time monitoring of exercise intensity distribution, at least for the lower intensity domains (Gronwald and Hoos, 2019; Gronwald et al., 2020a; Rogers and Gronwald, 2021; Rogers et al., 2021a,b).

### Modifiable Non-Exercise-Related Factors

As shown in Figure 1, there are several different sources contributing to the observed interindividual variability in a specific outcome parameter. Thus, in addition to exercise-related factors, also the influence of modifiable non-exercise related acute and chronic factors (e.g., diet, sleep, environmental factors, psychological stress, motivation, fatigue, or recovery status) needs to be considered. In this context, there is some evidence that there are individual differences in response to diets (Ordovas et al., 2018; Hughes, 2019; Morand and Tomás-Barberán, 2019; Zeisel, 2020), to sleep e.g., affected by sleep patterns (Nedelec et al., 2018) or to sleep loss (Tkachenko and Dinges, 2018). Hence, it is not surprising that considerable efforts have been undertaken to individualize nutritional interventions (de Roos and Brennan, 2017; Hughes, 2019; Zeisel, 2020) or sleep interventions (Nedelec et al., 2018; Walsh et al., 2020c). However, the influence of diet and sleep on interindividual response variability in neurocognitive outcome parameters after an acute bout of physical exercises has, to the best of our knowledge, not been investigated so far.

In addition, environmental influencing factors (e.g., climatic and geographic conditions) and the actual state of the level of psychophysiological capacity (including abilities like cardiorespiratory fitness/performance level) should also be considered to compare the effects of acute physical exercise regarding interindividual response variability (Gronwald and Budde, 2019; Herold et al., 2019; Gronwald et al., 2020b). For example, it has been observed that the effects of acute physical exercises on cognitive performance (Chang et al., 2012; Oberste et al., 2019) or functional brain activation patterns (Li et al., 2019; Ludyga et al., 2019; Cui et al., 2020) are modulated by the level of cardiorespiratory fitness or by regular physical activity level.

Moreover, there is evidence in the literature (i) that environmental factors (e.g., acute hypoxia) influence cognitive performance (Taylor et al., 2015; McMorris et al., 2017; Martin et al., 2019; Ando et al., 2020), (ii) that there are considerable interindividual differences in acute and chronic responses (e.g., cardiorespiratory response hemoglobin mass) to this factors (e.g., acute and chronic hypoxia) (Chapman et al., 1998; Friedmann et al., 2005; Chapman, 2013; Divert et al., 2015; Nummela et al., 2021), and (iii) that there is some degree of interindividual variability concerning acute physical and cognitive performance in environmental challenging conditions (e.g., hypoxia) (Walsh et al., 2020b). Interestingly, Walsh et al. (2020b) noticed that a higher hemoglobin concentration at high altitude was associated with a decline in cognitive performance in digit symbol substitution task following exercise at high altitude, which suggest a potential neurobehavioral relationship between individual physiological responses and changes in cognitive performance. However, the interaction of acute physical exercises and environmental factors (e.g., hypoxia and temperature) on interindividual variability in neurocognitive outcomes has, so far, not been extensively studied and needs further well-designed investigations to draw robust conclusions.

Collectively, these findings support the idea that the individual level of capacity of several abilities and individual differences in physiological responses are, among others, factors that contribute to the observed interindividual response variability. However, we have only an inchoate knowledge about what exact proportion of interindividual response variability is explained by the level of capacity or physiological responses (e.g., in relation to other modifiable non-exercise-related factors such as nutrition or sleep).

Summarizing the evidence presented in this section, it seems reasonable to assume that modifiable non-exercise-related lifestyle and personal factors can contribute to the interindividual response variability in the outcome parameter of interest in the context of acute physical exercises. However, we still have only an incomplete understanding to which exact extent these modifiable non-exercise-related personal lifestyle and environmental factors can explain the interindividual response variability in neurocognitive outcome parameters after an acute bout of physical exercises. This, in turn, calls for further research in this direction.

#### Recommendations Regarding Modifiable Non-Exercise-Related Factors

We recommend the following strategies which enable investigators to account, at least for a certain extent, for the influence of modifiable non-exercise-related factors: (i) test the physical performance level appropriately and consider the information from the test for exercise prescription, (ii) inform and ask the participants to follow specific behaviors (e.g., advise the participant to not consume ergogenic nutritional substance or maintain adequate sleep habits within the study period), and (iii) use questionnaires (e.g., to assess long-term diet habits or sleep quality, sport-specific training background, and physical training age) and/or activity trackers (e.g., to measure regular physical activity level and sleep) to control for changes within the study period. Such data (e.g., quality of sleep and sport-specific training background) can be used in the statistical analysis (e.g., as covariates) to quantify their contribution to the observed interindividual variability in a neurocognitive outcome parameter of interest (see section *Considerations regarding statistical analysis*). From a practical point of view, it should be kept in mind that it is difficult to consider and quantify every possible modifiable non-exercise-related factor, and thus it is a valid approach to focus on the most influential ones being important to answer the specific research question(s).

Moreover, we recommend that future studies should assess additional psychological factors (e.g., level of arousal, mood, and expectations) to elucidate the sources of interindividual response variability (Boot et al., 2013; Beedie et al., 2018; Green et al., 2019; Lindheimer et al., 2019a,b). In this regard, it has been observed that acute exercise-induced changes in arousal levels are linked to changes in functional cortical hemodynamics (Byun et al., 2014), whereas others found that expectation effects are an unlikely explanation for most of the variance in acute physical exercise-induced changes in cognitive functions (Oberste et al., 2017). In order to examine a possible influence of psychological factors, the use of a placebo (sham) group that performs the same exercise but without loading or without a target-specific dose may be helpful (Beedie et al., 2018; Budde et al., 2018; Green et al., 2019; Herold et al., 2020a,c). In this context, an appropriately designed control condition (CC; e.g., sham group that exercises without loading) can also help to “blind” the participants although complete blinding of the participant in acute exercise intervention studies is hardly possible as the participant is aware if she/he is exercising (or not) and has, at least a subjective feeling, at which exercise intensity she/he is exercising (Ludyga et al., 2016; McSween et al., 2019). While the complete blinding of the participants is not possible, we recommend, in accordance with others, the blinding of the personnel contributing to the study (e.g., assessor and data analyst), the rigorous standardization of test procedures (e.g., standardized encouragement), and to withheld information on the hypothesized efficacy of the acute physical intervention from the participants (if ethically possible) in order to minimize the risk of bias (Hecksteden et al., 2018a).

### Other Influencing Factors

Individual changes in specific outcome parameters have often been investigated as a response to a specific physical exercise intervention. Here, repeated measurements on the same subject are needed (e.g., measurements prior to and after an acute bout of physical exercises). Even if the gold standard method is used, some measurement error and biological variability are unavoidable so that a certain extent of the observed interindividual response variability is caused by these above-mentioned factors (Atkinson and Nevill, 1998; Atkinson and Batterham, 2015; Hopkins, 2015; Swinton et al., 2018; Atkinson et al., 2019; Ross et al., 2019; Bonafiglia et al., 2021a). Hence, the understanding of the measurement error and biological variability (both components are summarized in the term *typical error*; Hopkins, 2000) is important to make trustworthy interferences or assumptions about the inter- and intraindividual variability (Atkinson and Batterham, 2015; Williamson et al., 2017; Atkinson et al., 2018; Bonafiglia et al., 2021a). In particular, typical error has two main components: (i) systematic bias (e.g., learning effects and difference in machine calibration) and (ii) random error (e.g., intraindividual variability – e.g., caused by biological day-to-day variations). Theoretical backgrounds are discussed in more detail elsewhere (Atkinson and Nevill, 1998; Hopkins, 2000). Indeed, intraindividual variability in cognitive performance is frequently reported in the cognition literature in general (MacDonald et al., 2006) and in the exercise-cognition literature in particular (Wu et al., 2011; Moore et al., 2013; Bento-Torres et al., 2019; Kao et al., 2019).

The understanding of intraindividual response variability bears a great potential for a better understanding of the interindividual response variability (Hecksteden et al., 2015, 2018b; Voisin et al., 2019; Chrzanowski-Smith et al., 2020). In this respect, it was shown that for physiological outcomes (e.g., citrate synthase maximal activity, protein content, and capillary density), the repetition of exactly the same intervention leads to a relatively high degree of intraindividual variability in response to exactly the same physical exercise stimulus which, in turn, can bias the assessment of interindividual response variability (Islam et al., 2021).

It has to be acknowledged that there are several *other influencing factors* contributing to interindividual response variability, but an extensive discussion of all these factors is beyond the scope of this perspective article. Hence, we focus on two important ones, namely the time of day (circadian rhythms) and how often cognitive tests are performed (e.g., learning effect or habitation effect).

With regard to the circadian rhythm, it has been shown that the time of day influences cognitive performance considerably (e.g., due to peak periods of circadian arousal; Dijk et al., 1992; Blatter and Cajochen, 2007; Schmidt et al., 2007; Anderson et al., 2014; Burke et al., 2015; Hodyl et al., 2016; Iskandar et al., 2016). These findings suggest that the time of day at which the tests are performed should be standardized. Unfortunately, information regarding this factor has been not appropriately reported in available studies (Yamazaki et al., 2017, 2018; Schwarck et al., 2019) which support our claim of a more rigorous study design (or, at least, the reporting of study procedures).

With respect to repeated cognitive testing (CT), substantial learning effects have been observed in various cohorts when the same cognitive tests are performed several times (Calamia et al., 2012). Indeed, it has been observed that a considerable degree of intraindividual day-to-day difference regarding the effects of acute physical exercises on distinct outcome measures (e.g., accuracy) is present, which could be, at least partly, attributed to habituation effects (Donath et al., 2017; Pontifex et al., 2019). In order to control for these learning effects, comparisons of the effects of different experimental and control interventions are recommended (Green et al., 2014, 2019).

All in all, these findings suggest that *other influencing factors*, such as measurement error, time-of-day, and/or learning effects are serious confounding factors in acute exercise-cognition studies which have to be considered when interindividual variability is analyzed.

#### Recommendations Regarding Other Influencing Factors

**Figure 2:**
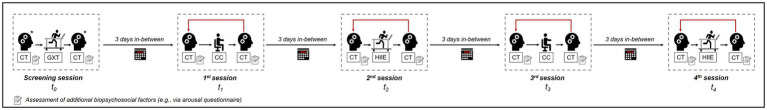
Schematic illustration of the study design “within-subject repeated (double) crossover design with pre-post-test comparisons” recommended to investigate the effects of an acute bout of physical exercises on cognitive performance. Please note that the order of the exercise and control conditions should be randomized. To investigate, for example, the effects of exercise intensity (high intensity vs. moderate intensity), two additional sessions would have to be added. In this context, we recommend to compare high-intensity interval exercise (HIIE) with moderate-intensity interval exercise (MIIE). If it is aimed to receive more details in regard to the relationship between the amount of exercise intensity and cognition (e.g., U-shaped relationship), at least three exercise intensities should be compared (e.g., light-, moderate-, and high-intensity interval exercise). The sessions t0, t1, t2, and t3, and t4 are separated each by 3 days (note: the relatively short duration between the sessions allows a standardization of the menstrual cycle phase for female participants with the inclusion of 4–5 sessions in the follicular phase and still ensure a sufficiently long period for recovery after e.g., high-intensity physical exercise; Elliott-Sale et al., 2021). ^*^indicates that cognitive testing during the initial session can be used to adequately habituate participants with the cognitive tests and testing protocol. CT, cognitive testing; CC, control condition; GXT, graded exercise test (e.g., running on a treadmill); HIIE, high-intensity interval exercise (i.e., running).

A crucial step to account for *other influencing factors* is the study design. The advantages and disadvantages of study designs in the context of interindividual response variability have been discussed in more detail elsewhere (Ross et al., 2019). The most rigorous study design in the acute exercise-cognition setting is the *within-subject repeated (double) crossover design with pre-post-test comparisons*. When using this design (see Figure 2) participants perform the cognitive test before and after the intervention condition (e.g., exercise condition or control condition) and participate on separate days in both the exercise condition(s) and the control condition, whereby the order is counterbalanced across the participants (crossover). The strengths of this design are (i) the reduction of potential subject-related confounds (e.g., genotype) as the participants serve as their own control, (ii) the possibility to account for possible day-to-day variations in the reference measure of cognitive performance (e.g., baseline shift) that can arise from the variety of influencing factors such as habituation (learning) effects and/or changes in psychological confounders (e.g., motivation), (iii) the assessment of the influence of measurement error (e.g., using the data of sham control condition that performs, for instance, a seated rest while watching a non-arousing video or performs a sham exercise with an insufficient loading), and (iv) the assessment of intraindividual variability as each condition is conducted twice (repeated/double crossover). A disadvantage of the *within-subject repeated (double) crossover design with pre-post-test comparisons* is the repeated conduction of the cognitive testing that might leads to pronounced learning effects/ceiling effects.

However, in consideration of the above-mentioned strengths of the *within-subject repeated (double) crossover design with pre-post-test comparisons* (for a detailed discussion from a statistical viewpoint, see Senn, 2016), we strongly recommend that acute exercise studies aiming to investigate the interindividual response variability in neurocognitive parameters use this study design (for example, see Figure 2).

In addition, to further reduce the influence of learning effects, an adequate familiarization with the testing procedures is recommended and the detailed steps of these procedures should be transparently reported (e.g., *When was a plateau in performance reached?* or *Which level of accuracy was accepted?*; McMorris, 2016; Donath et al., 2017). This point is particularly important because we know that a considerable (day-to-day) habitation effect occurs with respect to changes of cognitive performance (e.g., change scores of executive functions) in response to an acute bout of aerobic exercises (Donath et al., 2017; Pontifex et al., 2019). Another approach to account for learning/habituation is to model the learning effect by using the data of a control group that performed repeated testing (e.g., by determining *z*-scores as done by Walsh et al., 2020b or by calculating linear-mixed effects models).

Of note, our conclusions concerning the effect of acute physical exercises on neurocognitive outcomes typically rely on the comparison of exercise condition and control condition. Thus, close attention should be paid to an appropriately designed control condition. An important function of the control condition is, for instance, to control for possible time effects. Consequently, it is a common standard to use equal time intervals in exercise condition(s) and the control condition (e.g., seated rest; Pontifex et al., 2009; Chang et al., 2011; Budde et al., 2012; Basso et al., 2015; Hwang et al., 2016; Ludyga et al., 2017; Yamazaki et al., 2018). For example, in study of Schwarck et al. (2019), the total duration of the experimental sessions varied considerably between the exercise and the control groups. In particular, while the exercise groups performed treadmill running for 35 min (including warm-up and cool-down), the control group was sitting for only 10 min. Because of the time difference of 25 min between exercise groups and control group, the interpretation of the results of this study is somewhat hampered. Hence, we propose to apply rigorous study designs so that solid conclusions about the interindividual variability in neurocognitive outcomes in response to acute physical exercises can be drawn.

In this regard, the selection of an appropriate control condition is of uttermost importance because our conclusions regarding acute exercise-related effects on cognitive performance are commonly based on statistical comparisons of an exercise to a control condition (Green et al., 2014; Pontifex et al., 2019). Currently, no general consensus has been achieved on the issue of which is the most appropriate control condition but it is undoubted that an active control condition is favorable (Green et al., 2014, 2019). In this context, it is important to emphasize that we define *active control condition* as a control condition in which the experimenter has contact with the participants (*contact-control condition*; Green et al., 2014). Thus, *active* does not refer to the level of physical activity in the control condition. This means that physically passive activities such as being seated at rest are included in term active control given the experimenter has contact with the participant. In exercise cognition research, the control condition commonly consists of (i) seated rest coupled with a non-arousing activity, such as reading, listening to an audio recording, or watching a video, or (ii) performing another physical exercise (sham exercise) which do not pose an adequate dose to induce considerable effects (e.g., stretching or toning exercise, performing exercises with no load) (Green et al., 2014; Pontifex et al., 2019). In general, an appropriate control condition should aim to isolate the characteristics of interest (e.g., exercise-related neurobiological processes leading to the improvement of cognitive performance after an acute bout of physical exercises) while minimizing the influence of confounders (e.g., socioemotional changes in motivation) (Green et al., 2014, 2019; Pontifex et al., 2019). In this context, important factors such as the research question/aim and the research setting, (e.g., ecological valid setting such as school) need also to be considered (Green et al., 2014, 2019; Pontifex et al., 2019) and thus no universal recommendation concerning the most appropriate control condition can be provided. However, a relatively straightforward approach to isolate the effects of acute physical exercises on neurocognitive parameters (at least in a laboratory-based setting) is to perform the “control activity” in both the control condition and the exercise condition (e.g., watching a non-arousing video while being in seated rest and while cycling).

In summary, researchers in this field are strongly encouraged to use a rigorous study design to draw solid conclusions regarding the interindividual response variability (e.g., of neurocognitive outcome parameters).

#### Considerations Regarding the Statistical Analysis

An important part of the analysis of interindividual response variability is the classification of responsiveness (e.g., being a responder or non-responder). This point has been extensively discussed in more detail elsewhere, and several methods have been proposed to analyze interindividual variability (Atkinson and Batterham, 2015; Hecksteden et al., 2015, 2018b; Bonafiglia et al., 2016, 2018, 2019, 2021a; Swinton et al., 2018; Atkinson et al., 2019; Voisin et al., 2019; Dankel and Loenneke, 2020a,b; Tenan et al., 2020; Padilla et al., 2021; Sainani et al., 2021). In this context, others prefer a characterization of responsiveness using a more statistical viewpoint (e.g., probability or likelihood of response; Walsh et al., 2020a,b). However, although currently no general consensus has been reached regarding a classification approach, a dichotomization, such as being a responder vs. being a non-responder, seems to be a non-favorable strategy for most outcome parameters as by doing so we fail to understand the “50 shades of responders” (e.g., the contribution of the different determinants that causes interindividual response variability; Senn, 2018; Atkinson et al., 2019). Hence, sophisticated analysis methods need to be applied in further studies. This, in turn, necessitates a stronger collaboration between exercise scientists and statisticians and/or data scientists (e.g., to use artificial intelligence, such as machine learning algorithms, Bayesian interference, or account for the effect of RTM) which has been recently called for Sainani et al. (2021). However, although appropriate statistical analysis is undoubtedly a crucial aspect to derive solid conclusions, it cannot offset the limitation arising from a poor study design which, in turn, supports our claim to use a rigorous study design.

Additionally, it should be noted that the responsiveness is outcome parameter specific (Pickering and Kiely, 2018) which means that there can be an improvement in one parameter (e.g., favorable change in cerebral hemodynamic response) while another parameter has not changed (e.g., behavioral performance). In this context, further research should also address the practical relevance of interindividual differences in cognitive performance in response to an acute bout of physical exercises in more detail. In this regard, the size of the test battery (conducting several cognitive tests) could be of interest as it allows to calculate latent variables [an unobserved (latent) variable calculated based on the performance in a specific set of cognitive tests] and/or a composite score which can be of importance to draw further conclusions. The advantages and disadvantages of such an approach and further methodological aspects regarding cognitive testing (e.g., near and far transfer effect) are discussed elsewhere in more detail (Green et al., 2014, 2019; Liu-Ambrose et al., 2018; Moreau and Wiebels, 2021). In this context, there is some evidence in the literature that cognitive tests showing reliable effects at the group level are not well-situated to detect individual differences (Draheim et al., 2021). Thus, the development of new cognitive tests being better suited for interindividual variability research can be a promising avenue to further elucidate interindividual differences in cognitive outcomes in response to acute physical exercises.

## Conclusion

The investigation of the interindividual variability of outcome parameters (e.g., neurocognition) in response to physical exercises is undoubtedly an important research field when one considers the need to develop efficient, physical exercise-based interventions to promote neurocognitive health. Given that several factors contribute to the phenomenon of interindividual response variability (shown in Figure 1), a rigorous study design is mandatory to draw solid conclusions about the influence of a single factor (e.g., exercise variables such as exercise intensity). Hence, we recommend that future studies in the research field of exercise-cognition should pay stronger attention to a more rigorous study design (shown in Figure 2), taking into account several factors that may have an influence on the results. Here, further studies should also consider, in particular, the effects of exercise prescription according to external load, internal load, and influencing factors in order to understand the interindividual response variability in neurocognitive outcome measures (Gronwald and Budde, 2019; Herold et al., 2019, 2020b,c; Gronwald et al., 2020b).

## Data Availability Statement

The original contributions presented in the study are included in the article/supplementary material, further inquiries can be directed to the corresponding author.

## Author Contributions

FH and TG wrote the first draft of the article. AT, DH, HB, LZ, TS, and NM reviewed and edited the drafted versions. All authors contributed to the article and approved the submitted version.

### Conflict of Interest

The authors declare that the research was conducted in the absence of any commercial or financial relationships that could be construed as a potential conflict of interest.
